# Echocardiographic two-dimensional speckle tracking identifies acute regional myocardial edema and sub-acute fibrosis in pediatric focal myocarditis with normal ejection fraction: comparison with cardiac magnetic resonance

**DOI:** 10.1038/s41598-020-68048-5

**Published:** 2020-07-09

**Authors:** Marcello Chinali, Alessio Franceschini, Paolo Ciancarella, Veronica Lisignoli, Davide Curione, Paolo Ciliberti, Claudia Esposito, Alessia Del Pasqua, Gabriele Rinelli, Aurelio Secinaro

**Affiliations:** 1 Department of Pediatric Cardiology and Cardiovascular Surgery, Bambino Gesù Children’s Research Hospital-IRCSS, Piazza Sant’Onofrio 4, 00165 Rome, Italy; 2 Department of Imaging, Bambino Gesù Children’s Research Hospital-IRCSS, Rome, Italy

**Keywords:** Heart failure, Cardiomyopathies

## Abstract

The aim here was to describe the role of speckle tracking echocardiography (STE), in identifying impairment in systolic function in children and adolescents with focal myocarditis and without reduction in ejection fraction. We describe data from 33 pediatric patients (age 4–17 years) admitted for focal myocarditis, confirmed by cardiac magnetic resonance (CMR), and without impaired ejection fraction and/or wall motion abnormalities. All children underwent Doppler echocardiography examination with analysis of global (G) and segmental longitudinal strain (LS) and CMR for the quantification of edema and myocardial fibrosis. Reduction in LS was defined according to age-specific partition values. At baseline, impaired GLS was present in 58% of patients (n = 19), albeit normal ejection fraction. LS was also regionally impaired, according to the area of higher edema at CMR (i.e. most impaired at the level of the infero-lateral segments as compared to other segments (*p* < 0.05). GLS impairment was also moderately correlated with the percentage edema at CMR (r = − 0.712; *p* = 0.01). At follow-up, GLS improved in all patients (*p* < 0.001), and normal values were found in 13/19 patients with baseline reduction. Accordingly persistent global and regional impairment was still observed in 6 patients. Patients with persistent LS reduction demonstrated residual focal cardiac fibrosis at follow-up CMR. Both global and regional LS is able to identify abnormalities in systolic longitudinal mechanics in children and adolescents with focal myocarditis and normal ejection fraction. The reduction in LS is consistent with edema amount and localization at CMR. Furthermore, LS identifies regional recovery or persistent cardiac function impairment, possibly related to residual focal fibrosis.

## Introduction

The incidence of overt myocarditis with impaired contractile function in children is estimated around 1 per 100,000 children per year^1^. In contrast, incidence of pediatric focal myocarditis with normal ejection fraction (EF) might be significantly underestimated, as in children and adolescents, clinical presentation is limited to non-specific chest pain^2^. Accordingly, when symptoms are mild and non-specific, children not always undergo ECG tracing and testing for laboratory troponin levels in the Emergency Room^3,4^. In addition, when cardiac evaluation is performed, abnormalities might not be evident on ECG and no significant abnormalities in regional and/or global systolic abnormalities might be found on echocardiography^3,4^. In fact, since in focal myocarditis edema is located in the epicardial layer of the ventricular wall, only cardiac magnetic resonance (CMR) permits its direct detection^5^.

In severe forms of myocarditis, endomyocardial biopsy (EMB) with Dallas diagnostic criteria, is suggested^6^. However, when left ventricular (LV) EF is normal and symptoms are transient (i.e. focal myocarditis), EMBs are usually not performed, especially in children due to the inherent procedural risk of this invasive procedure^7^. CMR has been used as a non-invasive diagnostic tool, offering a 79% accuracy rate, when applying strict diagnostic criteria^8^. However, accessibility of CMR is often limited and thus it might not always be performed in children with suspected myocarditis. Furthermore, in younger children limited patient compliance might require sedation to perform the study. Eventually, evaluation of focal myocarditis using CMR requires substantial experience, which might not be available in all clinical settings^9^. Consequently, diagnosis and risk stratification of acute focal myocarditis by an objective measure remains a clinical challenge in pediatric setting. Standard 2D echocardiography is often the preferred imaging test, but its diagnostic accuracy is significantly limited. In contrast, quantitative measurement of regional myocardial deformation by Speckle Tracking Echocardiography (STE), may offer better sensitivity than conventional echocardiography for the detection of subclinical LV dysfunction and it may therefore improve the diagnostic and prognostic accuracy in acute focal myocarditis patients^10–12^. This might be particularly relevant in children with normal conventional echocardiographic parameters of the left ventricle.

Our aim was to evaluate the ability of STE in identifying acute abnormalities in systolic function occurring during focal myocarditis (confirmed by at least one revised CMR Lake Louise criteria) in children with normal EF and no evident wall motion abnormalities. In addition, our secondary aim was to evaluate whether STE might have a role in identifying patients with persistent subclinical systolic dysfunction at follow-up, possibly due to residual myocardial fibrosis.

## Methods

### Study population

**Figure 1:**
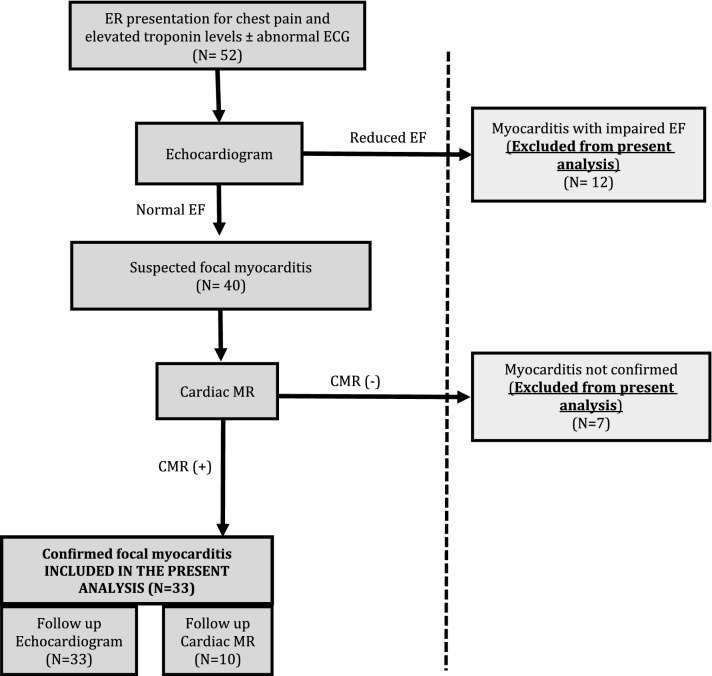
Study flow chart.

As shown in Fig. 1, data from consecutive patients from July 2013 to February 2018, admitted for chest pain associated with elevated troponin levels were retrospectively analyzed.

Exclusion criteria for the present analysis were: (1) Abnormal admission 2D echocardiography defined as: presence of regional wall motion abnormalities and/or reduced EF (< 55%) and/or more than mild pericardial effusion; (2) No cardiac edema and/or fibrosis found at baseline CMR. Among 52 patients admitted for chest pain associated with elevated troponin levels, 12 were excluded for reduced EF with wall motion abnormalities at baseline echocardiogram and 7 were excluded due to baseline CMR negative for myocarditis. All of the 7 patients excluded due to negative CMR, had reported intense physical activity performed before the occurrence of chest pain, possibly justifying the presence of mildly increased troponin levels in the absence of myocarditis. Of note, all patients excluded due to negative CMR had normal both EF (68.4 ± 5.5%) and GLS (–24.9 ± 3.0%). Accordingly, the study included 33 patients, comprising 25 boys and 8 girls (age 2–17 years: median 13 years [IQR 10–16]).

According to our clinical protocol, patients were admitted to the hospital for clinical observation, laboratory testing with troponin level curve and viral PCR analysis, serial ECG evaluation, complete echocardiography examination and CMR.

Echocardiographic evaluation was performed at admission and discharge. CMR for the analysis of myocardial edema and fibrosis, was performed at admission. Patients with confirmed focal myocarditis and impaired global (G) longitudinal strain (LS), where asked to volunteer for a follow-up CMR performed 4 ± 1 weeks after discharge. Among patients with evident impaired GLS, 53% agreed to perform a follow-up CMR.

The study was approved by the “Ospedale Pediatrico Bambino Gesù Ethics Committee” and the investigation conformed to the principles outlined in the Declaration of Helsinki. Written informed consent was obtained from a parent and/or legal guardian.

### Echocardiography

All patients underwent complete transthoracic echocardiographic examination with commercially available machines (Epiq7 or iE33, Philips Heathcare Inc.). Examinations were analyzed off-line on dedicated workstations (QLab Cardiac Analysis ver.10, Philips Heathcare Inc.) by two expert independent readers blinded of the clinical data (AF, MC). Two-dimensional images were obtained for the analysis of LV volumes on three consecutive beats from apical 4- and 2-chamber views. Wall thickness and chamber dimensions were obtained from the two-dimensional parasternal long axis or M-mode short axis at the midventricular level, when perfect alignment of the left ventricle was possible, on three consecutive beats^13^.

Parameters measured in our study included LV diameters and wall thicknesses to obtain LVM. LV mass was calculated according to Devereux’s formula^13^. LV mass was analyzed both as unindexed and as indexed by age-specific allometric powers. To define LV hypertrophy, a partition value of 45 g/(m^2.16^ + 0.09) was used^14^. For evaluation of LV geometry, myocardial thickness (wall + septum) was divided by LV minor axis (diameter) to generate a relative wall thickness (RWT) normalized to a mean age of 10 years old. A value of 0.38 was used as the cutoff to define concentric LV geometry^15^.

LV systolic function was determined by LV EF calculated using the biplane Simpson formula. Normal EF was defined according to current adult guidelines with a cut-off of 55%^16^. Standard Doppler analysis was performed to obtain LV inflow velocities at the mitral valve tips, including peak early diastolic filling (E) and late diastolic peak velocities (A).Tissue Doppler analysis was performed to obtain longitudinal early diastolic (E′) velocities at both the septal and the lateral was in order to derive the mitral E/e′ ratio^17^.

Strain analysis was used to obtain regional and global longitudinal strain (GLS) of the left ventricle on three consecutive beats from each apical window (apical 4-chamber, 3-chamber and 2-chamber window)^18^, comprising a total of 9 beats for each GLS analysis. In details, when adequate echocardiographic examinations were available (defined as images with good image quality and frame rate > 50 frames per second), feature tracking of the myocardium was achieved through the combination of echocardiographic tracking of speckle signals, mitral annulus motion, tissue-blood border detection, and periodicity of the cardiac cycle using R–R intervals. Accuracy of border tracking was visually confirmed by viewing the cardiac cycle with only border information displayed. If necessary, individual regions of the border were adjusted until the border was correctly tracked for each frame. Cardiac strain was used to calculate myocardial deformation and displayed it in a 17-segment model according to AHA guidelines^15,17^. Averaged segmental peak strain were calculated to obtain regional and global longitudinal strain. The software provides peak orthogonal deformation of the cardiac muscle during contraction, accordingly as in systole the myocardium reduces its wall length in the longitudinal planes, more negative values of longitudinal strains represent a better cardiac contraction. Intra- and Inter-observer variability for STE analysis was also tested.

Prevalence of subclinical cardiac systolic dysfunction was defined by previously reported age-specific strain partition values obtained from a meta-analysis of over 1,100 children, with impaired GLS defined as GLS > −20.5% in age range 2–9; GLS > −19.1% in age range 9–13; and GLS > −19.2 in age range 14–21^19^.

### Cardiac magnetic resonance

CMR studies were performed with a Siemens Aera 1.5-T scanner with 32-element coil (Siemens, Erlangen, Germany). CMR at baseline was performed for analysis of cardiac ejection fraction, and for the identification of acute edema, inflammatory damage and/or myocardial necrosis and at follow-up to rule out cardiac fibrosis. CMR was considered positive for focal myocarditis if at least one modified Lake-Louis criteria was satisfied^8^.

CMR images were acquired at end-expiration, applying a vector-ECG triggering. Imaging protocol in all patients included cine steady-state free precession (SSFP) sequences acquired on the short axis (SAX) from base to apex and long axis cardiac planes: FOV 400 × 310 mm^2^, slice thickness 7 mm, no interslice gap, acquisition matrix 256 × 173, voxel size 1.5 mm × 1.5 mm × 7 mm, echo/repetition time (TE/TR) 1.1/40 ms, readout bandwidth 930 Hz/pixel, flip angle 69°, 25 phases.

Myocardial edema was assessed using a T2-weighted triple inversion recovery black blood sequence, on the same planes of cine SSFP sequences, with the following parameters: FOV 400 × 290 mm^2^, slice thickness 7 mm, acquisition matrix 256 × 156, voxel size 1.3 × 1.3 × 7, inversion time (TI) 180 ms, TSE factor 25, bandwidth 504 Hz, half scan factor 0.65, TE 100 ms, TR 2 RR intervals for heart rates ≤ 80/min or 3 RR for heart rates > 80/min and 2 signal averages.

Late gadolinium enhancement (LGE) imaging was performed using a phase sensitive inversion recovery (PSIR) T1-weighted gradient echo pulse sequence, acquired along the same planes previously described, 10 min after intravenous administration of contrast agent (Gadoterate meglumine—DOTAREM, Roissy, Guerbet, France) at 0.2 mmol/kg [FOV 400 × 290 mm^2^, acquisition matrix 256 × 156, slice thickness 7 mm, voxel size 1.3 × 1.3 × 7, repetition time 747 ms, echo time 3 ms, flip angle 25°, inversion time (TI) ranging from 280 to 400 ms. The correct TI for nulling the signal of healthy myocardium was identified with a look-locker sequence acquired in SAX plane].

Analysis of the images previously acquired was performed with an off-line workstation (CMR42, Circle Cardiovascular Imaging, Calgary, AB, Canada). Cine short axis stack was analyzed using the modified Simpson’s method of disk summation. Left ventricle end-diastolic volume (EDV), end-systolic volume (ESV), end-diastolic mass (EDM) and ejection fraction (EF) were calculated for each patient. EDV, ESV and EDM have been also indexed for body surface area (BSA) with the Mostseller formula.

Tissue characterization was performed with a semi-automatic quantification of myocardial edema and LGE on the SAX stacks, applying a threshold of 2- and 5 standard deviations (SD) above the mean values of the remote myocardium, respectively. Left ventricle endocardial and epicardial borders were manually drawn in every SAX image. Remote myocardium was also manually identified tracing a myocardial reference region of interest on the healthy muscle in every slice.

### Statistical analysis

Data for continuous variables are presented as means ± standard deviation, while categorical variables are presented as proportions or percentages. Continuous variables were tested by Kolmogorov–Smirnov to confirm normal distribution. Non-normally distributed parameters are presented as median ± interquartile range. When needed for multivariate analysis, non-normally distributed parameters were log-transformed for testing. Comparison of normally distributed continuous variables, between baseline and follow-up, was performed by Student's paired *t* test and by Chi-square for categorical data. Comparison of non-normally distributed continuous variables was performed by Mann–Whitney *U* test. Correlation between variables was evaluated by linear regression analysis (for continuous variables) or logistic regression (for categorical variables), when appropriate. A *p* value < 0.05 was considered statistically significant. Statistical analysis was performed by SPSS software, version 22.0 (IBM Corporation, Armonk, NY, USA).

## Results

### Clinical characteristics

As detailed in Table 1, clinical evaluation at admission revealed: chest pain in all patients (33/33), history of flu-like symptoms within 8-weeks before admission in 24/33, palpitation in 5/33 patients.

**Table 1:** Clinical characteristics of study population at admission (n = 33).

	Mean or percentage	N or SD
Gender (M)	76%	[N = 25]
Age (years)	16 [Median]	IQR [10–16]
Flu-like symptoms	73%	[N = 24]
Chest pain	100%	[N = 33]
Palpitations	15%	[N = 5]
ECG abnormalities (of which):	60%	[N = 20]
- ST elevation	60%	[N = 12]
- Non-specific abnormal T wave	40%	[N = 8]
Peak Troponin (ng/ml)	1,051 [Median]	IQR [5–9]
Viral PCR (positive)	11/33	33%
Heart rate (bpm’)	106	± 23
SBP (mmHg)	108	± 18
DBP (mmHg)	64	± 12

Baseline ECG showed abnormalities in 20/33 patients, including ST-segment elevation in 12/33 and non-specific T wave abnormalities in 8/33. Laboratory testing at admission demonstrated increase in inflammation markers and cardiac enzymes in all patients. All patients were free from any other concomitant disorder (except one patient with known Klinefelter syndrome).

Mean hospital stay was 5 ± 3 days. All patients were in good hemodynamic status during hospital stay and no episodes of cardiac arrhythmia were detected. Patients were discharged when free of symptoms and with evidence of normalized both troponin levels and ECG tracings. No specific medication was prescribed in any of the patients except Ibuoprofen 10 mg/kg T.I.D. in patients who presented concomitant pericardial effusion (n = 7).

### Baseline echocardiographic findings

Both intra- and inter-observer variability analyses showed good reproducibility of GLS data with high interclass correlation coefficients (IntraCC = 0.91 [IC95% 0.76–0.97] and InterCC: (ICC of 0.88 [IC95% 0.72–0.91]).

As shown in Table 2, all patients had normal EF at admission (EF 61 ± 5%) and no evident wall motion abnormalities.

**Table 2:** Echocardiographic characteristics of study population at admission (n = 33).

	Mean	SD or N
LVIDD (cm)	4.6	0.6
LV mass index (g/m^2.16^ + 0.09)	37.7	8.3
Ejection Fraction (%)	61.0	5.5
Transmitral E/A ratio	1.7	0.4
Mitral E/e′	6.1	2.3
Pericardial effusion (cm)	0.2	0.1
Mitral regurgitation (mild/moderate/severe)	3/0/0	–
Global longitudinal strain (%)	− 19.7	2.5
Impaired global longitudinal strain (%)	58%	N = 19

LV geometry was normal in all patients with no evidence of LV hypertrophy and/or dilation. Pattern of diastolic filling was also in the normal range for age, with no evidence of increased LV filling pressure (defined as E/e′ > 12). Seven patients (or 21%) presented pericardial effusion, which was mild in all cases (max 0.4 cm). In contrast, prevalence of clear-cut reduced global longitudinal strain was present in 58% of patients (n = 19) who showed mildly reduced global longitudinal strain (− 18.1 ± 1.7 vs. − 22.1 ± 1.3; *p* < 0.001). Reduction in longitudinal strain was not uniformly distributed in all left ventricular segments, but was most frequently found at the infero-septal, inferior and infero-lateral segments.

Of note, in 15 of the 19 patients with normal global longitudinal strain, isolated regional LS values over − 19% could be observed, suggesting that despite normal GLS, mild reduction in regional strain could be identified in overall 29 of the 33 studied patients (88%).

Thus, reduction in longitudinal strain was related to the areas of CMR-identified edema. In particular, accordingly to the distribution of the CMR edema, most patients showed significantly reduced regional longitudinal strain in the area comprising infero-septal, inferior and infero-lateral walls (i.e. IL sector; GLS = −16.2 ± 5.5%) and as compared to the mean of the other segments (− 23.5 ± 4.2%; *p* < 0.05) (Tables 3 and 4).

**Table 3:** Clinical and echocardiographic characteristics of the study population dichotomized by normal VS impaired longitudinal strain.

	Normal N = 14	Impaired N = 19	P value
Age (years)	13.7 ± 2.8	12.7 ± 3.9	0.434
Peak Troponin I (ng/mL)	3,023 ± 5,227	9,137 ± 19,248	0.910^a^
LVM index (g/m^2.16^ + 0.09)	35.4 ± 9.2	38.2 ± 8.7	0.123
Ejection fraction (%)	61.7 ± 5.7	60.5 ± 5.5	0.542
Transmitral E/A ratio	1.9 ± 0.36	1.7 ± 0.42	0.187
Mitral E/e′	6.1 ± 2.7	6.3 ± 3.7	0.347
Pericardial effusion; N of pts (cm)	N = 3 (0.2 ± 0.1)	N = 4 (0.2 ± 0.1)	0.874
Mitral regurgitation (mild/mod./sev.)	4/0/0	6/0/0	–
Global longitudinal strain (%)	− 22.1 ± 1.3	− 18.1 ± 1.7	< 0.001
Inferior macro sector^b^ LS% (%)	− 21.5 ± 4.2	− 16.2 ± 5.5	< 0.001
CMR-edema (g)	11.4 ± 8.9	26.2 ± 13.0	0.043
CMR-edema (%)	13.3 ± 6.7	26.8 ± 13.9	0.041

^a^Mann–Whitney *U* test.

^b^Inferior macro sector LS = LS%(Inferoseptal) + LS%(Inferior) + LS%(Inferolateral).

**Table 4:** Regional longitudinal strain in segments according to the presence of baseline normal versus impaired GLS%.

AHA 17-segment	Normal (N = 14)	Impaired (N = 19)	*p* value
**Basal segments**			
Basal anterior LS (%)	− 24.2 ± 4.1	− 21.1 ± 3.2	0.19
Basal anteroseptal LS (%)	− 19.7 ± 3.1	− 19.0 ± 4.6	0.33
Basal inferoseptal LS (%)	− **22.7 ± 3.3**	− **17.6 ± 3.7**	**0.02**
Basal inferior LS (%)	− **28.3 ± 4.0**	− **16.7 ± 2.8**	** < 0.001**
Basal inferolateral LS (%)	− **23.6 ± 4.2**	− **17.1 ± 3.1**	**0.01**
Basal anterolateral LS (%)	− 22.0 ± 3.8	− 18.5 ± 4.0	0.07
**Mid-ventricular segments**			
Mid anterior LS (%)	− 20.1 ± 2.7	− 23.4 ± 2.2	0.24
Mid anteroseptal LS (%)	− 21.5 ± 3.9	− 22.2 ± 3.5	0.45
Mid inferoseptal LS (%)	− 21.9 ± 3.2	− 18.5 ± 4.2	0.08
Mid inferior LS (%)	− **26.2 ± 2.8**	− **17.5 ± 3.6**	**0.01**
Mid inferolateral LS (%)	− **20.1 ± 2.9**	− **16.7 ± 3.0**	**0.04**
Mid anterolateral LS (%)	− 25.1 ± 3.7	− 23.1 ± 4.1	0.21
**Apical segments**			
Apical anterior LS (%)	− 23.6 ± 3.5	− 22.1 ± 3.3	0.62
Apical septal LS (%)	− 22.0 ± 3.9	− 20.5 ± 3.8	0.45
Apical inferior LS (%)	− 21.5 ± 3.7	− 20.5 ± 4.0	0.33
Apical lateral LS (%)	− 21.8 ± 3.3	− 21.7 ± 3.2	0.21
Apex LS (%)	− 23.6 ± 3.4	− 22.1 ± 3.1	0.51

The data in bold refers to statistically significant difference.

In regression analysis, a borderline association was found between reduction in global longitudinal strain and peak troponin I levels (r = −0.41; *p* = 0.05).

### Baseline CMR findings

**Figure 2:**
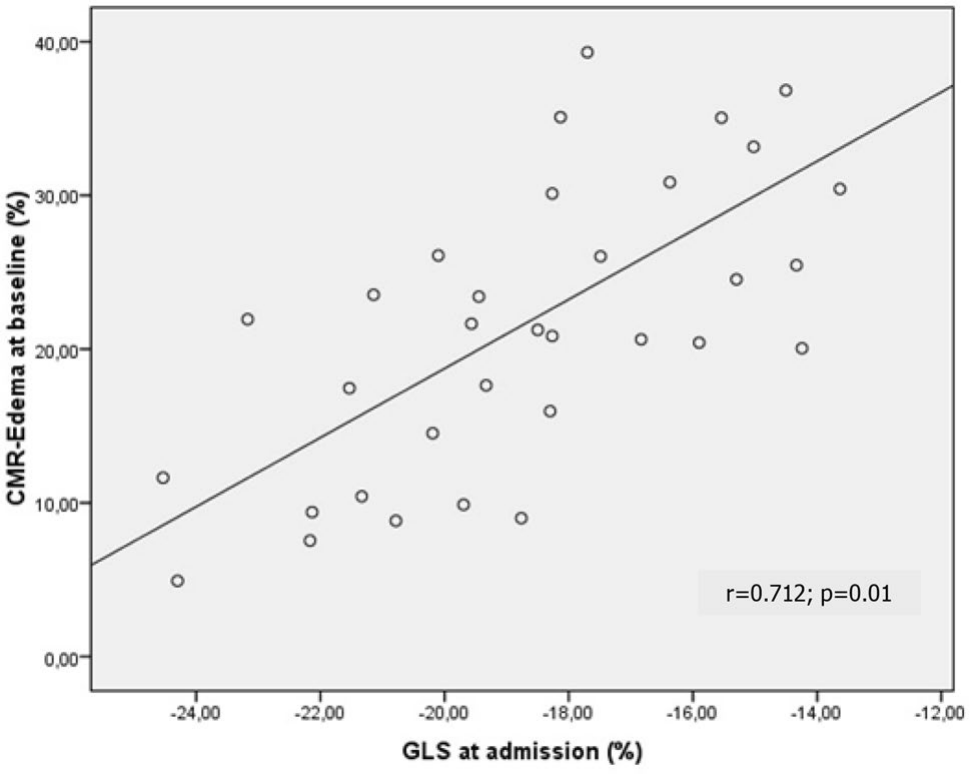
Regression between percent of edema at baseline MRI and GLS% at admission (see text from explanations).

Analysis of baseline CMR showed a mean EF of 59.4 ± 5.7%. In contrast to echocardiography, a mild reduction in EF could be observed in two patients (49% and 50% respectively), with no evident regional hypokinesia. Analysis of myocardial edema at presentation showed a mean edema of 22 ± 13 g of edema, representing a 20.9 ± 9.5% of edematous myocardial mass (range 4.9–39.3%). In regression analysis, a moderate association was found between percentage of edema at CMR and GLS at admission (r = 0.712; *p* = 0.01; see Fig. 2).

### Follow-up

**Figure 3:**
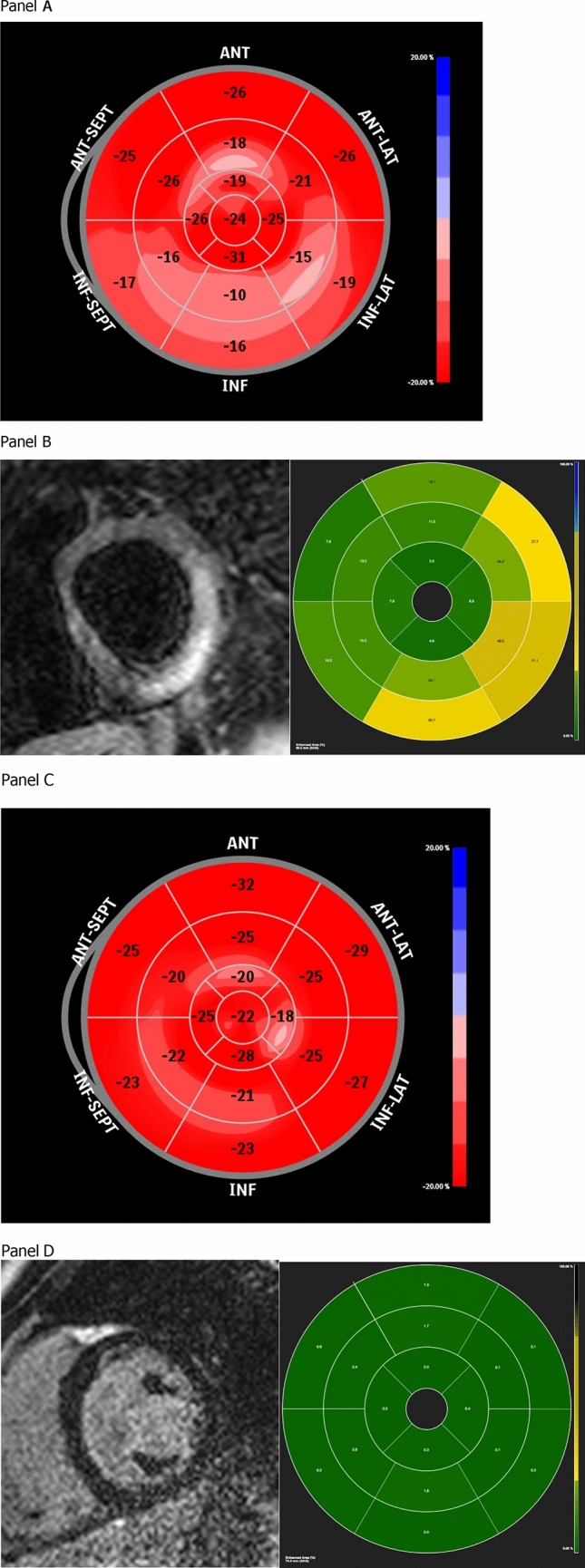
Example of patient with recovered systolic dysfunction at follow-up (Panel **A** and **C** images acquired with QLab Cardiac Analysis ver.10, Philips Heathcare Inc., Panel **B** and **D** images acquired with CMR42, Circle Cardiovascular Imaging, Calgary, AB, Canada). (**A**) Evidence of impaired GLS at baseline echocardiographic evaluation. (**B**) Evidence of significant focal edema at baseline CMR (qualitative and quantitative). (**C**) Evidence of recovered GLS at follow-up echocardiographic evaluation. (**D**) Evidence of no focal fibrosis at follow-up CMR (qualitative and quantitative).

Echocardiographic examination performed at follow up, showed no changes in EF as compared to baseline (*p* = ns). In contrast, a significant reduction in longitudinal strain could be observed in all children, with a mean improvement of 2.21% [range from 1.22 to 3.65 (*p* < 0.001)]. When analyzing regional variation in longitudinal strain, the highest improvement was observed at the infero-lateral segments (mean + 4.85%). As a result, the prevalence of clear-cut impaired GLS, decreased from 58 to 19% (*p* < 0.05), demonstrating full recovery in systolic function in 13 kids and persistent subclinical dysfunction in 6 patients (Fig. 3).

**Figure 4:**
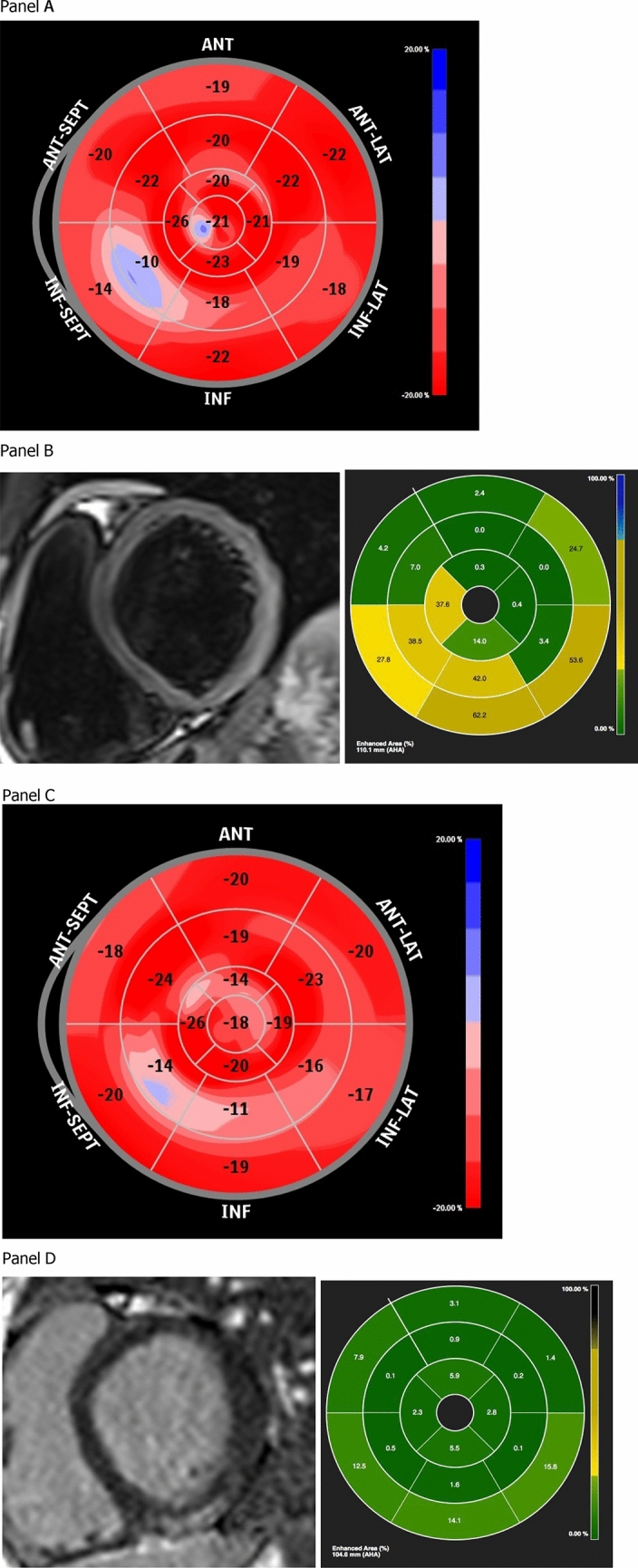
Example of patient with persistent systolic dysfunction at follow-up (Panel **A** and **C** images acquired with QLab Cardiac Analysis ver.10, Philips Heathcare Inc., Panel **B** and **D** images acquired with CMR42, Circle Cardiovascular Imaging, Calgary, AB, Canada). (**A**) Evidence of impaired GLS at baseline echocardiographic evaluation. (**B**) Evidence of significant focal edema at baseline CMR. (**C**) Evidence of persistent impaired GLS at follow-up echocardiographic evaluation. (**D**) Evidence of residual focal fibrosis at follow-up CMR.

Of note, in the 6 patients with persistent dysfunction, peak troponin I at admission was significantly higher as compared to patients with evidence of subsequent strain normalization (Median: 19,252 vs. 1,994 pg/mL; Mann–Whitney *U* test *p* = 0.023). At follow-up, 10 patients among the 19 with impaired GLS at baseline underwent a second voluntary CMR. Of these, 4/10 had persistent dysfunction at discharge echocardiography while GLS had recovered in 6. Patients with persistent dysfunction demonstrated higher cardiac fibrosis at LGE analysis (Fig. 4), as compared to patients with full recovery at follow-up echocardiography, in which little or no LGE could be observed (− 15.8 ± 9.8 vs. − 2.9 ± 4.1; Mann–Whitney *U* test *p* = 0.014), demonstrating higher presence of post-myocarditis myocardial fibrosis in patients without GLS recovery at follow-up (Table 5).

**Table 5:** Changes in echocardiographic variables in the study population from hospital admission to discharge in children according to global longitudinal strain class at admission.

	Normal (n = 14)			Impaired GLS (n = 19)		
	Admission	Discharge	*p* <	Admission	Discharge	*p* <
EF (%)	61.7 ± 5.7	62.5 ± 5.2	0.55	60.5 ± 5.5	61.7 ± 5.7	0.54
E/A ratio	1.8 ± 0.33	1.7 ± 0.25	0.47	1.9 ± 0.36	1.7 ± 0.42	0.18
Transmitral E/e′	6.2 ± 1.4	6.0 ± 1.7	0.71	6.1 ± 2.7	6.3 ± 3.7	0.34
Per. effusion (mm)	2 ± 1 mm	0 mm	0.42	2 ± 1 mm	0 mm	0.67
MR (+/++/+++)	4/0/0	4/0/0	–	6/0/0	6/0/0	–
GLS (%)	− 22.1 ± 1.3	− 23.5 ± 1.2	0.06	− 18.1 ± 1.7	− 21.8 ± 2.1	0.01
IMS-LS(%)	− 21.5 ± 4.2	− 23.2 ± 1.3	0.12	− 16.2 ± 5.5	− 20.2 ± 1.5	0.04
Impaired GLS n (%)	0 (0%)	0 (0%)	0.99	19 (58%)	6 (18%)	0.03

IMS-LS% = mean [LS%(Inferoseptal) + LS%(Inferior) + LS%(Inferolateral)].

## Discussion

Our main findings is that in children and adolescents with focal myocarditis and normal EF, 2-dimensional STE identifies subclinical abnormalities in systolic function, consistent with the regional presence of CMR-epicardial edema. In addition, analysis of STE provides insights in lack of regional recovery in systolic function during follow-up, possibly related to the presence of residual myocardial fibrosis.

Acute myocarditis is characterized by viral damage on the cardiac muscle, which might be either direct or mediated by an immune reaction. The immune-relate myocardial damage is usually mediated by the action of T-lymphocytes and antibodies, which are directed against both pathogens and endogenous heart epitopes. The immune response resulting from this reaction involves all of the myocardial tissue, although usually without clinical sequels^20^.

Accordingly, in the acute phase of myocarditis, cardiac necrosis markers (as troponin I) are consistently increased, and thus have been found to have high specificity (89%) in the diagnosis of otherwise clinically silent focal myocarditis^21^. In our study, all patients presented with significantly increased heart necrosis biomarkers; however, we found only a very mild correlation between TnI and GLS (*p* = 0.05). This is in partial agreement with previously reported findings from adults^11,12^, and it has been speculated that one of the possible explanation for the partial lack of correlation between TnI and GLS in focal myocarditis is that the inflammation caused by acute myocarditis does not necessarily induce proportionate myocytolysis^11^. Thus, the amount of myocardial lysis does not necessarily reflect the amount of cardiac edema^22^, which in turn is the main determinant of LS impairment, as also demonstrated by our current findings. Also, given the focal nature of myocarditis in our population, GLS might not accurately reflect the regional impairment found in cardiac muscle. Of note, the Dallas pathological criteria for the diagnosis of myocarditis although requiring presence of inflammatory cellular infiltrate, do not consider myocyte necrosis a compulsory finding to confirm the diagnosis of acute myocarditis^8^.

Our findings might be of considerable clinical interest as the regional edema induced by myocarditis, does not usually lead to overt abnormalities in regional and/or global cardiac function and thus are usually not detected by conventional echocardiography, unless the percentage of cardiac edema is of considerable amount. However, especially in the setting of acute focal myocarditis, the amount of myocardial edema involvement is usually minor. Thus, in this setting the value of strain imaging might be of paramount importance as it allows identification of regional edema and resulting regional subclinical systolic dysfunction.

In agreement to previous studies performed in adults, global and regional myocardial deformation was reduced in a significant amount of our patients^11,12^. Also, the amount of edema was associated to the severity of GLS dysfunction, independently of echocardiographic EF^11^. This finding suggests that myocardial deformation analysis is more likely to detect subtle changes or mild myocardial damage as compared to traditional echocardiographic parameters of both systolic and/or diastolic function. In addition, we found that the reduction in LS was due to a regional reduction in systolic function, rather than in a global systolic impairment. The reduction in LS was mostly consistent with CMR findings in which presence of edema was mainly localized in the infero-postero-lateral segments.

Our findings suggest that analysis of regional and global LV myocardial deformation by LS in young population gives additional information on both the location and the degree of cardiac involvement in focal myocarditis as compared with conventional echocardiography. In particular, analysis of LS performed at admission provides reliable information on the location and severity of the myocardial edema occurring in children with acute myocarditis, without evident reduction in traditional indices of cardiac performance. In addition, analysis of LS at discharge suggested that the reduction in cardiac performance is reversible in most cases, as demonstrated by the normalization of GLS and regional values. However in a percentage of patient, GLS remains impaired also at follow-up, suggesting some degree of myocardial damage occurring in these patients beyond acute edema. This was confirmed, albeit only in a small subgroup of patients, when comparing data from follow-up CMR. In fact, late evidence of LGE (i.e. fibrosis) was significantly higher in children with persistent reduced GLS as compared to those in which GLS returned to normal values at follow-up echocardiogram. It is interesting to highlight that focal myocarditis is generally considered a benign, self-limiting event. However, our analysis suggests that despite no clinically evident cardiac impairment, focal myocarditis may lead to subclinical mid-term cardiac dysfunction in a relevant number of children (18% in our study sample).

The main limitation of our study relies on the small study sample included in the analysis. This limitation is even more relevant regarding the follow-up CMR, which was in fact available only in 10 out of 33 patients. It should be noted however, that in children CMR often requires general anesthesia and thus, giving the limited evidence of clinical relevance of repeated CMR in focal myocarditis in children with normal EF, the follow-up CMR could be included in our study protocol, only if performed on a voluntary basis. Another intrinsic limitation of the study relies on in its retrospective design from a single-center.

Nonetheless, despite these limitations, our data supports that LS might be of use especially when repeated CMR is not performed, as it provides accurate data on residual fibrosis, thus providing additional information for risk stratification in this clinical setting.

In conclusion, our data suggest that analysis of cardiac function by two-dimensional STE, provides useful diagnostic information in pediatric acute myocarditis with normal EF. CMR, remains the gold standard and should be always considered in the diagnostic test of pediatric patients with suspected acute myocarditis. However, when CMR is not available or requires total anesthesia, analysis of advanced parameters of cardiac mechanics by STE, might provide important surrogate information.

## References

[1] Dancea AB (2001). Myocarditis in infants and children: a review for the paediatrician. Paediatr. Child. Health.

[2] Cooper LT, Keren A, Sliwa K, Matsumori A, Mensah GA (2014). The global burden of myocarditis: part 1: a systematic literature review for the Global Burden of Diseases, Injuries, and Risk Factors 2010 study. Glob. Heart.

[3] May LJ, Patton DJ, Fruitman DS (2011). The evolving approach to paediatric myocarditis: a review of the current literature. Cardiol. Young.

[4] Levi D, Alejos J (2001). Diagnosis and treatment of pediatric viral myocarditis. Curr. Opin. Cardiol..

[5] Kühl U, Schultheiss HP (2010). Myocarditis in children. Heart Fail. Clin..

[6] Chow LH, Radio SJ, Sears TD, McManus BM (1989). Insensitivity of right ventricular endomyocardial biopsy in the diagnosis of myocarditis. J. Am. Coll. Cardiol..

[7] Cooper LT, Baughman KL, Feldman AM, Frustaci A, Jessup M, Kuhl U (2007). The role of endomyocardial biopsy in the management of cardiovascular disease. A scientific statement from the American Heart Association, the American College of Cardiology, and the European Society of Cardiology. Endorsed by the Heart Failure Society of America and the Heart Failure Association of the European Society of Cardiology. J. Am. Coll. Cardiol..

[8] Friedrich MG, Sechtem U, Schulz-Menger J, Holmvang G, Alakija P, Cooper LT, White JA, Abdel-Aty H, Gutberlet M, Prasad S, Aletras A, Laissy JP, Paterson I, Filipchuk NG, Kumar A, Pauschinger M, Liu P (2009). International consensus group on cardiovascular magnetic resonance in myocarditis. J. Am. Coll. Cardiol..

[9] Lurz P, Eitel I, Adam J, Steiner J, Grothoff M, Desch S (2012). Diagnostic performance of CMR imaging compared with EMB in patients with suspected myocarditis. JACC Cardiovasc. Imaging.

[10] Khoo NS, Smallhorn JF, Atallah J, Kaneko S, MacKie AS, Paterson I (2012). Altered left ventricular tissue velocities, deformation and twist in children and young adults with acute myocarditis and normal ejection fraction. J. Am. Soc. Echocardiogr..

[11] Løgstrup BB, Nielsen JM, Kim WY, Poulsen SH (2016). Myocardial oedema in acute myocarditis detected by echocardiographic 2D myocardial deformation analysis. Eur. Heart J. Cardiovasc. Imaging.

[12] Di Bella G, Gaeta M, Pingitore A, Oreto G, Zito C, Minutoli F, Anfuso C, Dattilo G, Lamari A, Coglitore S, Carerj S (2010). Myocardial deformation in acute myocarditis with normal left ventricular wall motion—Sa cardiac magnetic resonance and 2-dimensional strain echocardiographic study. Circ. J..

[13] Devereux RB, Alonso DR, Lutas EM, Gottlieb GJ, Campo E, Sachs I, Reichek N (1986). Echocardiographic assessment of left ventricular hypertrophy: comparison to necropsy findings. Am. J. Cardiol..

[14] Chinali M, Emma F, Esposito C, Rinelli G, Franceschini A, Doyon A, Raimondi F, Pongiglione G, Schaefer F, Matteucci MC (2016). Left ventricular mass indexing in infants, children, and adolescents: a simplified approach for the identification of left ventricular hypertrophy in clinical practice. J. Pediatr..

[15] de Simone G, Daniels SR, Kimball TR, Roman MJ, Romano C, Chinali M, Galderisi M, Devereux RB (2005). Evaluation of concentric left ventricular geometry in humans: evidence for age-related systematic underestimation. Hypertension.

[16] Lang RM, Badano LP, Mor-Avi V, Afilalo J, Armstrong A, Ernande L, Flachskampf FA, Foster E, Goldstein SA, Kuznetsova T, Lancellotti P, Muraru D, Picard MH, Rietzschel ER, Rudski L, Spencer KT, Tsang W, Voigt JU (2015). Recommendations for cardiac chamber quantification by echocardiography in adults: an update from the American Society of Echocardiography and the European Association of Cardiovascular Imaging. Eur. Heart J. Cardiovasc. Imaging.

[17] Nagueh SF, Smiseth OA, Appleton CP, Byrd BF, Dokainish H, Edvardsen T, Flachskampf FA, Gillebert TC, Klein AL, Lancellotti P, Marino P, Oh JK, Popescu BA, Waggoner AD (2016). Recommendations for the evaluation of left ventricular diastolic function by echocardiography: an update from the American Society of Echocardiography and the European Association of Cardiovascular Imaging. J. Am. Soc. Echocardiogr..

[18] Amundsen BH, Helle-Valle T, Edvardsen T, Torp H, Crosby J, Lyseggen E, Støylen A, Ihlen H, Lima JA, Smiseth OA, Slørdahl SA (2006). Noninvasive myocardial strain measurement by speckle tracking echocardiography: validation against sonomicrometry and tagged magnetic resonance imaging. J. Am. Coll. Cardiol..

[19] Levy PT, Machefsky A, Sanchez AA, Patel MD, Rogal S, Fowler S, Yaeger L, Hardi A, Holland MR, Hamvas A, Singh GK (2016). Reference ranges of left ventricular strain measures by two-dimensional speckle-tracking echocardiography in children: a systematic review and meta-analysis. J. Am. Soc. Echocardiogr..

[20] Chang H, Hanawa H, Yoshida T, Hayashi M, Liu H, Ding L (2008). Alteration of IL-17 related protein expressions in experimental autoimmune myocarditis and inhibition of IL-17 by IL-10-Ig fusion gene transfer. Circ. J..

[21] Smith SC, Ladenson JH, Mason JW, Jaffe AS (2007). Elevations of cardiac troponin I associated with myocarditis: experimental and clinical correlates. Circulation.

[22] Aretz HT, Billingham ME, Edwards WD, Factor SM, Fallon JT, Fenoglio JJ, Olsen EG, Schoen FJ (1997). Myocarditis: a histopathologic definition and classification. Am. J. Cardiovasc. Pathol..

